# Supplementary material for: Quantification and Phenotypic Characterization of Extracellular Vesicles from Patients with Acute Myeloid and B-Cell Lymphoblastic Leukemia
Source: Cancers (Basel). 2021 Dec 23;14(1):56. doi: 10.3390/cancers14010056 (PMC8750511; doi:10.3390/cancers14010056)
Supplement: Supplementary file 1 [file cancers-14-00056-s001.zip › cancers-1503829-supplementary.pdf]

SUPPLEMENTARY INFORMATION

Miljkovic-Licina et al.

SUPPLEMENTARY MATERIAL AND METHODS

Cell lines

Kasumi-1 cells (obtained from DSMZ-German Collection of Microorganisms and Cell Culture GmbH) and JeKo-1 (a kind gift from J.A. Martinez-Climent; Laboratory of Molecular Oncology, Center for Applied Medical Research, University of Navarra, Madrid, Spain) were cultured in RPMI-1640 containing Glutamax-1 (Invitrogen, Carlsbad, CA, USA) supplemented with 20% fetal bovine serum (FBS; Life Technologies, Carlsbad, CA, USA). MV-11-4, BJAB, Jurkat (all a kind gift from R. Zubler, Hematology Service, Geneva University Hospital), and K562 (obtained from ATCC-American Type Culture Collection) were cultured in RPMI-1640 containing Glutamax-1 (Invitrogen), supplemented with 10% FBS. OCI-AML3 cell line (obtained from DSMZ) was cultured in minimal essential medium (α MEM; Invitrogen) supplemented with 20% FBS. MDA-MB-231 (breast adenocarcinoma cell line; a kind gift of Patrick Meraldi, University of Geneva) was cultured in Leibovitz's L-15 medium (Invitrogen) supplemented with 10% FBS.

Isolation and Purification of EVs from conditioned medium (CM)

To obtain maximal EV quantities from CM supernatants, different cell lines were cultured in serum-free medium for as long as the apoptotic rate at the end of culture remained < 5% (between 12 and 72h, depending on the cell line used). Culture supernatants were then recovered and cells, cell debris as well as larger vesicles were removed by serial centrifugations (300 x g for 10 minutes, 2,000 x g for 30 minutes, and 10,000 x g for 45 minutes), and by filtration through a 0.22 µm membrane (Millex-GV, Millipore Corp. Carrigtwohill, Co. Cork, Ireland). EVs were further isolated from these pre-cleared samples by total exosome precipitation reagent (Life Technologies, Invitrogen), resuspended in 25-100 µL of PBS, and kept at -80°C for long-term storage. Total protein was quantified in CM-derived EV preparations by the Micro BCA™ Protein Assay kit (Thermo Fisher Scientific).

Cryo-Electron Microscopy (Cryo-EM)

For direct visualization of vesicles by Cryo-EM, total EV pellets from PFP samples were resuspended in PBS and applied on lacey carbon film grids (300 microMesh, Electron Microscopy Sciences, Hatfield, PA, USA). The grid was blotted in an automatic plunge freezing apparatus Vitrobot mark IV (Thermo Fisher, Hillsboro, USA) to control humidity and temperature. Observations were made at -170°C in a Tecnai F 20 microscope (Thermo Fisher Scientific) operating at 200 kV and equipped with a cryo-specimen holder Gatan 626 (Gatan, Warrendale, PA, USA). Digital images were collected using a Falcon III camera (Thermo Fisher Scientific) with 4,096 X 4,096 pixels using different magnifications (29,000X with a pixel size of 0.29 nm and 50,000X with a pixel size of 0.2 nm). The defocus range was from -1.6 µm to -2.5 µm applying an electron dose of 30 $e^-/Å^2$.

Conventional Flow Cytometry Analysis

For analysis of cell lines by flow cytometry, cells were washed in CWB containing 2% BSA. Cell suspensions were then stained for 15-20 min at room temperature with CD9-APC (1:50; Miltenyi Biotec), CD63-APC (1:50; Miltenyi Biotec), CD81-APC (1:50; Miltenyi Biotec), CD34-PE (1:5; Beckman Coulter), CD105-PE (1:50; Miltenyi Biotec), CD44-FITC (1:5; BioLegend, clone IM7103022), SSEA-4-APC-Vio770 antibodies (1:10 ; Miltenyi Biotec) and IPS-K-4A2B8 (1:1; a kind gift from H-J. Bühring). Cells were washed and re-suspended in staining buffer containing 7-AAD (5 µL, Beckman Coulter) to exclude non-viable cells.

SUPPLEMENTARY TABLE

**Table S1: Patient characteristics**								
Patient	Age	Sex	Clinical diagnosis	Phenotype (not complete)	Cytogenomics	Molecular Biology	Blast counts	Thrombocytes
**AML**							(G/L)	(G/L)
1	72	F	AML NPM1pos	CD34pos CD33pos CD14 neg CD19neg	normal	NPM1 mut	309	33
2	66	M	AML with t(6;9)	CD34pos CD33pos CD14neg CD19neg	t(6;9)	DEK-NUP214	15	44
3	56	F	AML NPM1pos	CD34partial CD33pos CD14neg CD19neg	normal	NPM1 mut, FLT3-ITD	97	49
4	81	F	AML NOS	CD34pos CD33 weak CD14neg CD19neg	normal	n.d.	41	27
5	72	F	sec AML	CD34pos CD33pos CD14neg CD19neg	complex	ETV6 rearranged;	56	87
6	86	F	sec AML	CD34pos CD33pos CD14neg CD19neg	del 5q	n.d.	3.8	5
7	70	F	AML, NPM1 pos (relapse)	CD34pos CD33pos CD14neg CD19 neg	normal	NPM1 mut, FLT3-ITD, IDH2 mut	167	43
8	64	F	AML M0; NOS	CD34pos CD33weak CD14neg CD19neg	del 20	ASXL1 mut, RUNX1 mut, IDH1mut	83	240
9	83	F	sec AML	CD34pos CD33pos CD14neg CD19neg	n.d.	n.d.	6	177
10	85	F	AML M5; NOS	CD34neg CD33pos CD14pos CD19neg	n.d.	n.d.	24	12
11	50	F	AML NPM1pos	CD34neg CD33pos CD14neg CD19neg	normal	NPM1mut, FLT3-ITD, TET2 mut	43	53
12	44	M	AML NPM1 pos	CD34neg CD33pos CD14partial CD19neg	normal	NPM1 mut	50	n.a.
13	67	F	AMLwith dysplasia	CD34pos CD33pos CD14neg CD19neg	complex	EVI 1+	3	130
14	40	F	AML M4; NOS	CD34pos CD33pos CD14 pos/neg CD19neg	normal	KRAS; WT1+	88	25
15	50	M	AML M4; NOS	CD34partial CD33pos CD14neg CD19neg	del7; del12	KMT2A rearranged	72	101
16	70	F	sec AML	CD34pos CD33pos CD14neg CD19neg	complex	n.d.	14	65
17	90	F	sec AML	CD34pos CD33 weak CD14neg CD19neg	complex	WT1 +	87	41
**B-ALL**								
18	64	M	B-ALL, Phi neg	CD34pos CD33neg CD14neg CD19pos	t(1;19)	Phi neg	53	n.a.
19	18	M	B-ALL, Phi neg	CD34pos CD33pos CD14neg CD19pos	hypodiploid	TP53 mut, CNS2a mut	10	88
20	18	M	B-ALL, Phi-like	CD34pos CD33neg CD14neg CD19pos	n.a.	del IKZF1	13	6
* n.a. = not available								

Table S1: Acute leukemia patients: phenotypic characteristics and clinical diagnosis

SUPPLEMENTARY FIGURES

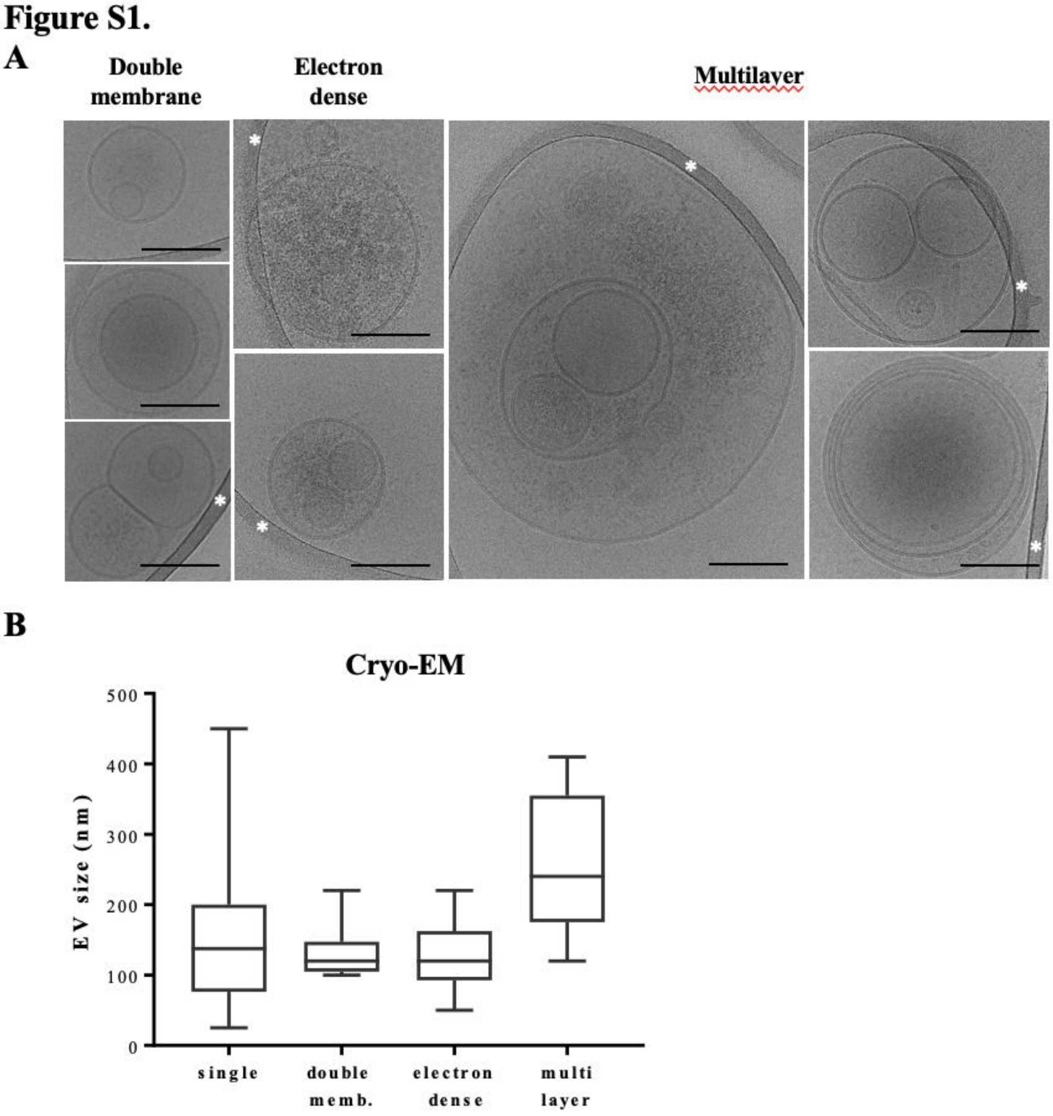

Figure S1: Cryo-EM

A) Electron micrographs of different forms of EVs observed by Cryo-EM: EVs with two lipid membranes, EVs with electron dense cargo; multilayer vesicles. Scale bar, 100nm. White asterisks mark strands of the supporting carbon net.

B) Size measurements (nm) of the different forms of EVs: single (n=26), double membrane (n=9), electron dense (n=10), and multilayered vesicles (n=5).

Figure S2.

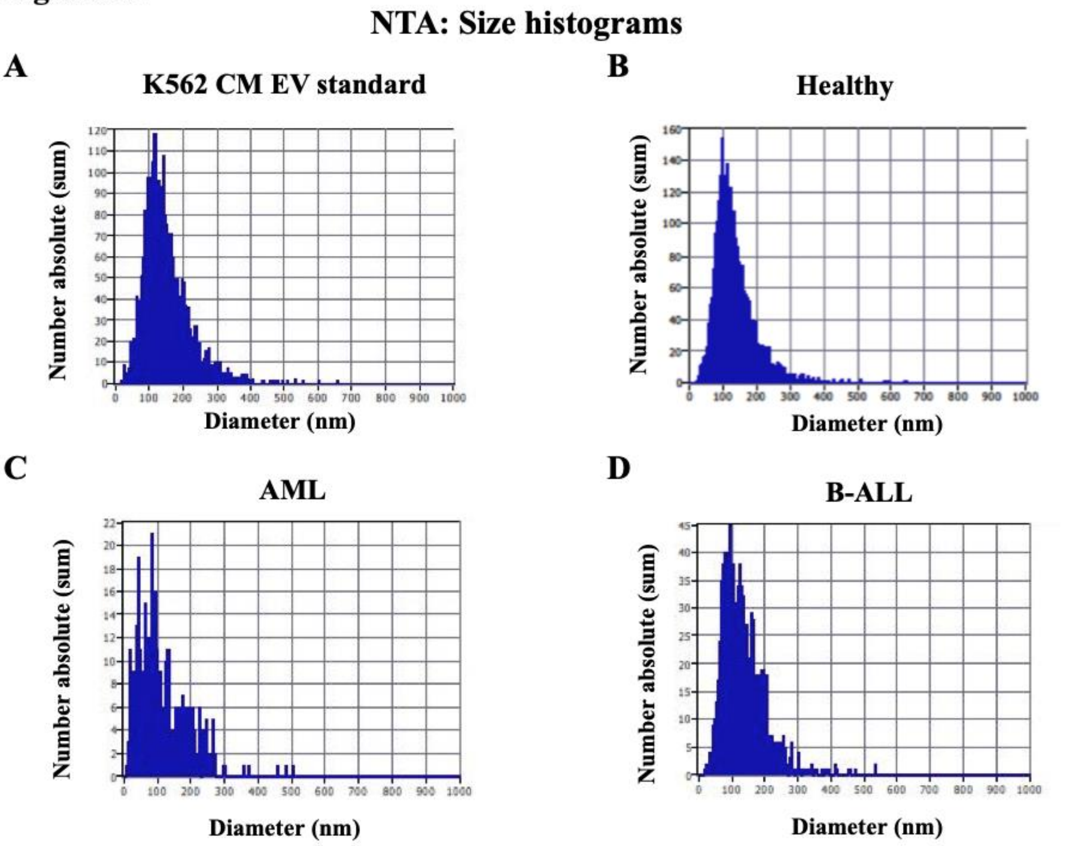

Figure S2.: NTA analysis of PFP samples

PFP samples were analyzed by NTA for the presence of detectable particles and representative size histograms are shown. EVs extracted from a supernatant of the K562 cell line were used as standard (Hansa BioMed Life Sciences). Size of particles detected in PFP samples from HBDs, AML and B-ALL patients did not differ significantly.

Figure S3.

A

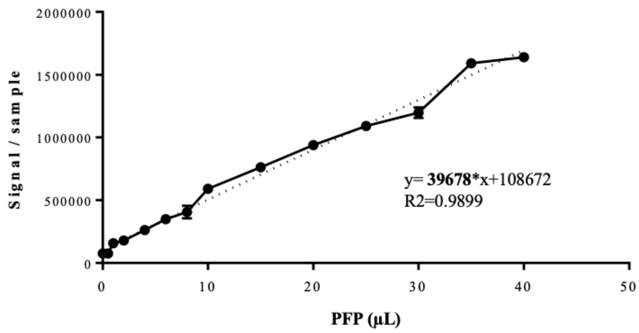

B

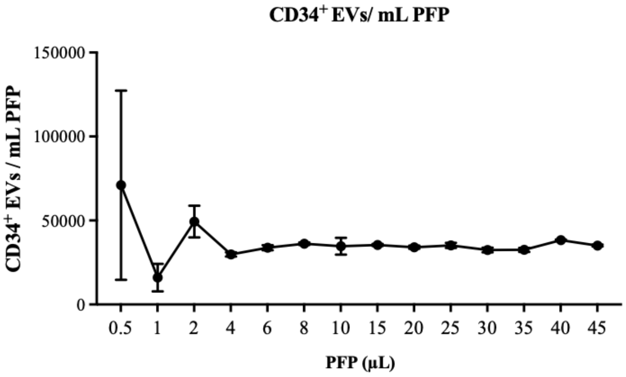

Figure S3: Determination of the saturating volumes of an AML PFP sample used for FT-FCM.

A) Titration curve representing mean fluorescent signal intensity detected by FL-2 triggering for a fixed amount of CD34-PE antibody (5 μL of stock solution) and various volumes of a PFP sample. For a constant quantity of CD34-PE antibody (5 μL), we increased the quantity of PFP sample from 0 to 45 μL. The signal intensities increased proportionally with the increase of PFP volumes confirming that the quantity of CD34 antibody was not limiting the PFP volumes tested. Dotted line represents the calculated regression line; R^2 = 0.99.

B) Quantification of $CD34^+$ EVs detected by FL-2 triggering based on measurements of various volumes of a representative PFP sample. Fixed concentration of anti-CD34 antibody (5 μL of stock solution) was used. The $CD34^+$ EV concentration/ sample was then determined by the slope of the regression line, e.g. 39'678 $CD34^+$ EVs/μL of PFP. As the $CD34^+$ EV concentration was the most variable for the smaller PFP volumes (i.e.: 0-8 μL), we used PFP volumes in the range from 10-30 μL for all further measurements.

Figure S4.

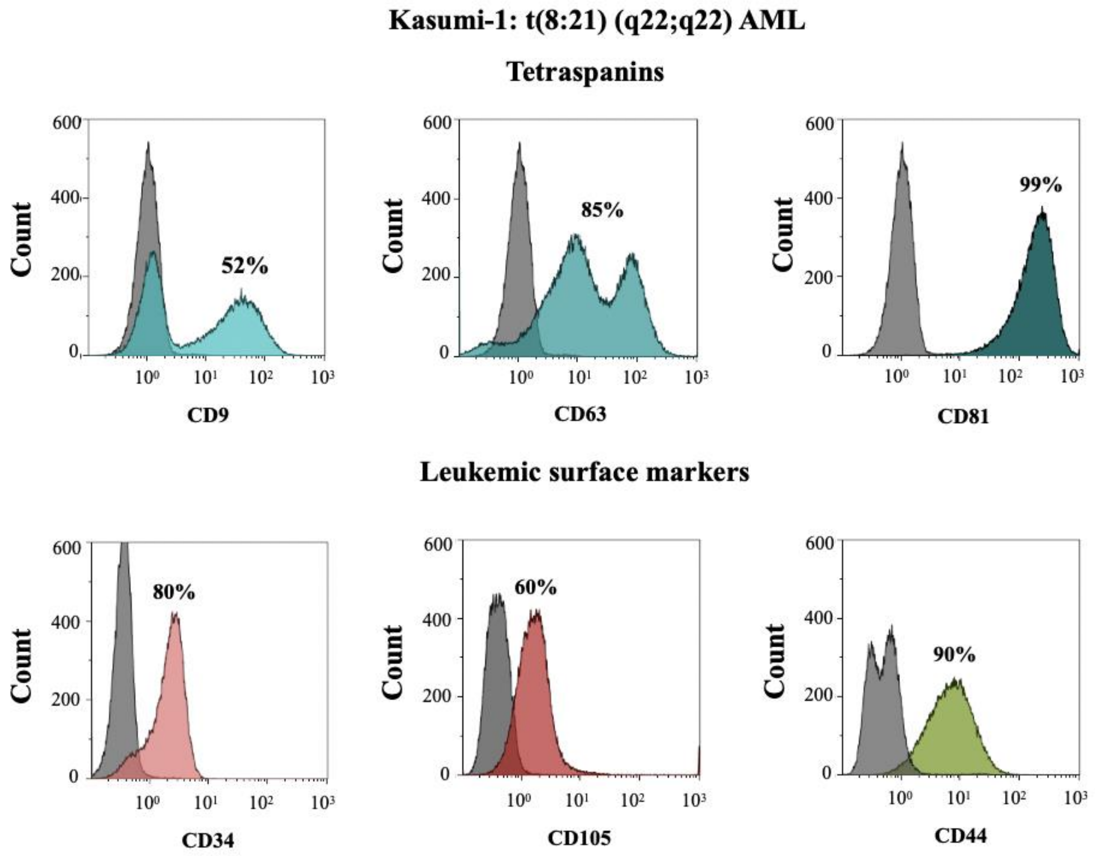

Figure S4: Flow cytometry

Conventional flow cytometry analysis of Kasumi-1 cells stained with CD9 (APC; light blue), CD63 (APC; blue), CD81 (APC; dark blue), CD34 (PE), CD105 (PE), and CD44 (FITC) antibodies, compared to respective unstained controls (dark grey).

Figure S5.

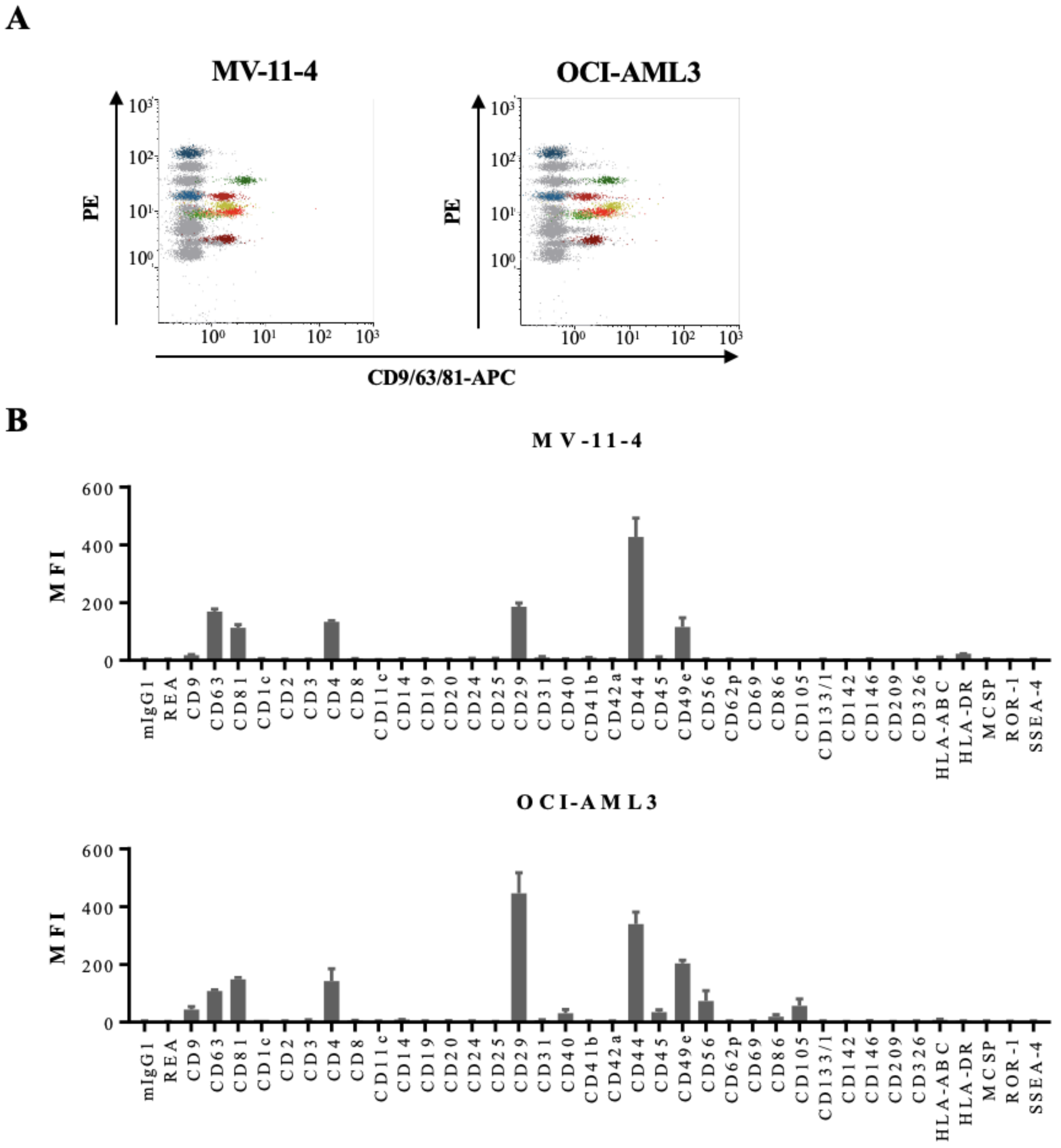

Figure S5: Multiplex bead-based flow cytometry assay on EVs derived from two leukemic cell lines.

Representative dot plots and quantification of multiplex bead-based flow cytometry analysis of MV-11-4 and OCI-AML3 CM-derived EVs showing measurements of single intensities of CD9/CD63/CD81-APC-stained 39 bead populations.

Figure S6.

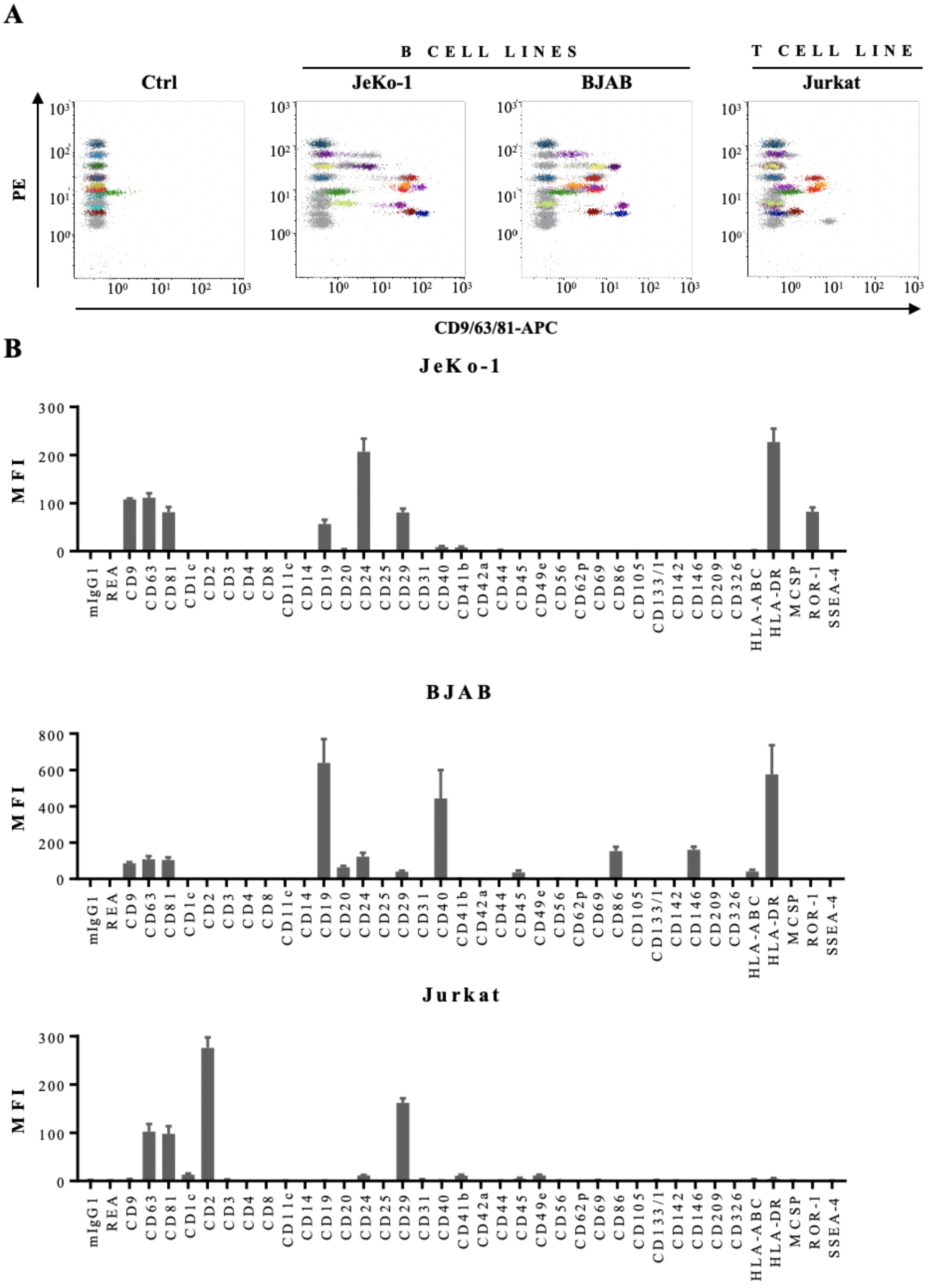

Figure S6: Multiplex bead-based flow cytometry assay on EVs secreted by B and T cell lines

A) Representative dot plots of multiplex bead-based flow cytometry analysis of CM-derived EVs isolated from two B cell lines (JeKo-1 and BJAB) and one T cell line (Jurkat), revealed with the CD9/CD63/CD81 antibody mix. Shown are measurements of single intensities compared to the respective serum-free medium control values (Ctrl, first panel).

B) Representative quantification of the median APC fluorescence intensities (MFI) for all bead populations after background correction.

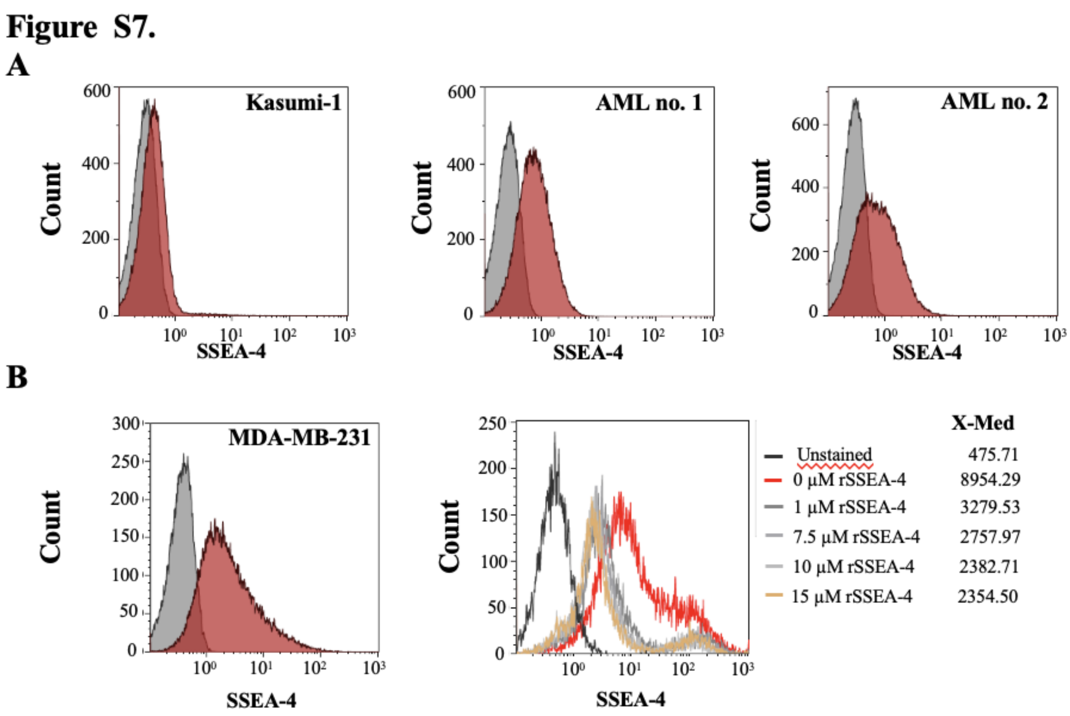

Figure S7: Flow cytometry of Kasumi-1 and primary AML cells

A) Flow cytometry analysis of Kasumi-1 cell line (negative control), MDA-MB-231 cell line (breast carcinoma; positive control), and two AML samples (patients 16 and 17; Table S1) stained with SSEA-4 antibody (APC-Vio770; red curve), compared to respective unstained controls (dark grey).

B) Specificity of staining was confirmed with IPS-K-4A2B8 monoclonal antibody against SSEA-4 using a positive control, MDA-MB-231 cell line and an inhibition assay with increasing concentrations of recombinant SSEA-4 antigen (Elicityl Oligotech).
